# High-Efficiency Traceability Mechanism for Multimedia Data in Consumer Internet of Things Combined with Blockchain

**DOI:** 10.3390/s26010074

**Published:** 2025-12-22

**Authors:** Tianyi Yan, Jimin Chen, Xiaorui Zhang, Gang Hu

**Affiliations:** 1 College of Computer and Information Engineering, Nanjing Tech University, Nanjing 211816, China; yantianyi@njtech.edu.cn (T.Y.); 0320257862@njtech.edu.cn (J.C.); zxr365@njtech.edu.cn (X.Z.); 2 School of Management Science and Engineering, Anhui University of Technology, Ma’anshan 243099, China

**Keywords:** Consumer Internet of Things, multimedia data, blockchain, PROV model

## Abstract

In the context of the rapid development of Consumer Internet of Things (CIoT), the manipulation and unauthorized distribution of multimedia content have raised serious concerns regarding copyright protection and data authenticity. Ensuring secure traceability and authenticity in complex network environments remains a major challenge. Traditional blockchain mechanisms often suffer from high latency during large-scale data queries, making them unsuitable for real-time CIoT applications. To address this, this paper proposes a high-efficiency blockchain-based multimedia data security traceability method. First, blockchain is integrated with the PROV model to guarantee operation transparency and data credibility. Second, a joint index structure comprising a fast index (using traceability positioning tables for cross-block jumps) and a multi-bucket index (using self-balancing binary trees) is designed. Experimental results demonstrate that compared to traditional blockchain and SEBDB methods, the proposed mechanism remains stable as data volume exceeds 2000 records. Specifically, the query time growth rate is significantly lower than linear scanning methods (12–18% vs. 45–62% for traditional methods), and the number of traversed records is reduced by 60–75% by avoiding full-chain traversal, verifying the method’s superiority in handling high-frequency CIoT multimedia queries while providing protection against tampering and unauthorized distribution.

## 1. Introduction

With the rapid proliferation of the Consumer Internet of Things (CIoT), billions of smart devices, wearable technologies, and home entertainment terminals are continuously generating massive volumes of multimedia data. This data plays a critical role in smart healthcare, intelligent education, interactive entertainment, and industrial applications. Notably, the growing prevalence of multimedia content tampering and unauthorized distribution has intensified concerns over copyright protection and ownership conflicts, drawing increasing attention from researchers across multiple fields. The unique characteristics of CIoT environments including device heterogeneity, resource constraints, and real-time processing requirements pose significant challenges for ensuring multimedia data security and integrity. In complex network environments, ensuring secure traceability, authenticity, and copyright protection for multimedia content has become a major challenge in CIoT applications. As a result, secure data traceability and trustworthy verification have become essential to address these challenges [1].

Data traceability ensures that every stage of data, from generation to consumption, can be accurately tracked and verified, guaranteeing its authenticity and integrity [2]. Specifically, data traceability not only tracks the source and flow of device data but also includes data processing stages, device status changes, and modification records [3]. Its primary goal is to maintain the integrity, credibility, and verifiability of data, allowing stakeholders to clearly understand the historical context and reliability of the data, thus reducing risks caused by information opacity, tampering, or forgery [4]. This traceability is particularly crucial in CIoT applications, where multimedia content is frequently interacted with [5]. However, traditional centralized traceability solutions are prone to single points of failure, failing to effectively prevent data tampering and copyright infringement [6,7]. These limitations become particularly problematic in CIoT environments where multimedia data requires real-time processing and high-frequency access, and where the distributed nature of devices makes centralized control impractical [8]. The key security challenges include: device heterogeneity, real-time processing requirements, content tampering risks, and the need for transparent traceability. Our proposed mechanism directly addresses these challenges through blockchain, PROV model, and optimized indexing. In recent years, blockchain technology [9,10], with its distributed, tamper-proof, and transparent characteristics, has provided a new approach for secure traceability of multimedia data in CIoT environments [11].

Blockchain, through its decentralized architecture, effectively avoids the risk of single points of failure, enhancing system robustness and data reliability. Additionally, cryptographic techniques such as hash algorithms and asymmetric encryption ensure the integrity and immutability of multimedia data across stages of collection, transmission, and storage, preventing malicious tampering or forgery [12]. Furthermore, the transparent distributed ledger allows authorized nodes to track data flow in real time, ensuring transparency and traceability of data sources and movements [13]. These characteristics make blockchain particularly suitable for addressing the security and traceability requirements of CIoT multimedia applications [14].

However, in CIoT environments with large-scale, frequently updated multimedia data, traditional blockchain data structures face significant performance bottlenecks. Research has shown that traditional blockchain systems struggle with efficiency in CIoT multimedia applications. Guo et al. [15] demonstrated the inefficiency of linear scanning in blockchain traceability, while Zhu et al. [16] highlighted limitations in existing blockchain databases. This limitation is particularly critical for multimedia applications that require real-time response and high throughput, such as video surveillance, live streaming, and interactive media services in CIoT environments. Moreover, the storage structure of traditional blockchains lacks optimization for multimedia content retrieval. The linear structure of conventional blockchains is ill-suited for the complex query patterns and rapid access requirements of multimedia data in CIoT applications. As a result, researchers have proposed solutions to build indexes on the blockchain. Data traceability often requires tracking across multiple blocks, and existing index designs are generally optimized for standard query needs, making them inadequate for the complex query requirements of multimedia data.

To address these fundamental challenges and provide an efficient, scalable solution for CIoT multimedia security, this paper proposes an efficient blockchain data traceability mechanism suitable for the CIoT multimedia security environment. It adopts a traceability framework based on blockchain and the PROV model to record the entire lifecycle operations of CIoT multimedia data, ensuring trustworthy and traceable data sources. Additionally, a joint index structure is designed, consisting of a fast index and a multi-bucket index. The fast index enables direct cross-block jumps, reducing block traversal and quickly locating the latest traceability block through the traceability positioning table. This cross-block efficient jump significantly improves query response speed. The multi-bucket index stores multimedia data objects in buckets based on keywords and uses a self-balancing binary search tree for efficient data management and retrieval. This approach effectively reduces the amount of data that needs to be scanned during queries while ensuring the efficiency of query operations. The proposed mechanism is specifically designed to overcome the performance limitations of traditional blockchain systems while maintaining the security benefits of blockchain technology for CIoT multimedia applications.

The main contributions of this study are as follows:

- A novel efficient blockchain-based multimedia data security traceability mechanism suitable for CIoT environments is proposed. This mechanism combines blockchain technology with the PROV model to ensure standardized recording of data operations and the credibility of data sources. A joint index structure, including fast indexing and multi-bucket indexing, is utilized to accelerate traceability queries while avoiding the introduction of external databases, improving data query efficiency, and maintaining blockchain security and transparency.
- A fast index structure optimized for CIoT multimedia application scenarios is designed. By storing key index items and combining them with the traceability positioning table, direct cross-block jumps can be achieved, significantly reducing block traversal operations. To further enhance the interpretability and decision-making value of the traceability path, a comprehensive path quantitative scoring method is proposed, evaluating the traceability path based on factors such as path length, block confirmation count, and node reputation.
- A multi-bucket indexing mechanism based on multimedia keywords is constructed to efficiently manage the grouping of data objects within blocks. This structure groups data based on multimedia data characteristics using keywords, and maintains the order and searchability of data within each bucket using a self-balancing binary search tree. Additionally, an instant adjustment algorithm is designed to address structural imbalance issues in high-concurrency environments, improving system stability without sacrificing query efficiency.
- The proposed approach comprehensively addresses key security challenges in CIoT multimedia applications, including protection against content tampering, unauthorized distribution, and ensuring data authenticity through cryptographic techniques and immutable blockchain records. The mechanism provides a robust framework for copyright protection and ownership verification in distributed CIoT environments.

The choice of blockchain technology for this research is motivated by its unique ability to provide decentralized trust, data immutability, and transparent audit trails all critical requirements for secure multimedia traceability in distributed CIoT environments. Unlike traditional centralized approaches, blockchain eliminates single points of failure and provides a tamper-resistant foundation for multimedia data management.

The rest of this paper is organized as follows: Section 2 introduces blockchain technology, related work on blockchain-based data traceability, and the current research on multimedia security in CIoT. Section 3 presents an efficient blockchain multimedia data traceability mechanism based on a joint index structure. Section 4 demonstrates the simulation experiments and result analysis, while Section 5 provides the conclusion of the paper.

## 2. Related Work

### 2.1. Blockchain Technology

**Figure 1:**
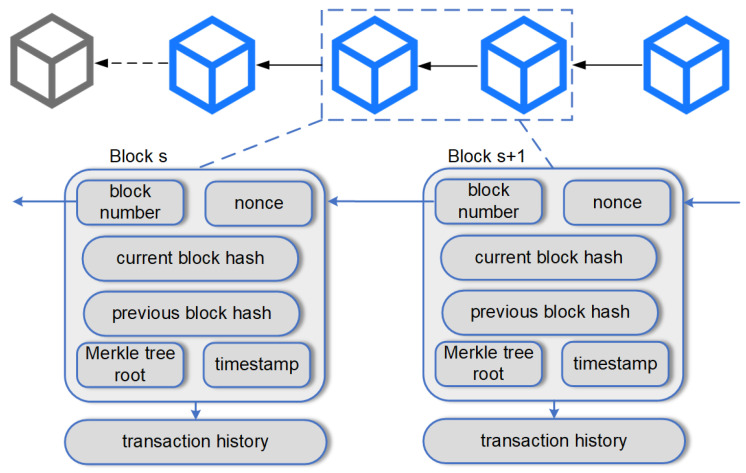
Blockchain data structure.

Blockchain technology is a distributed ledger technology based on the concept of decentralization [12], which aims to guarantee the security, transparency, and immutability of data storage using encryption algorithms and consensus mechanisms [17]. The core concept is to split data into multiple “blocks,” each containing several transaction records, which are linked to the previous block using an encrypted hash algorithm to create a chain structure [18]. In a blockchain network, all participating nodes have the same copy of the ledger, and the addition and update of data depends on the consensus of all nodes in the network. Common consensus mechanisms include Proof of Work (PoW) [19], Proof of Stake (PoS) [20], Byzantine Fault Tolerance (BFT) [21], etc. These mechanisms ensure the decentralized operation of the network in different ways, prevent single-point failures and ensure the security and fairness of the network [22]. Before each transaction is recorded on the blockchain, it must be encrypted and verified by the participating nodes. The legitimacy of the transaction is guaranteed by a specific algorithm to ensure that the transaction is tamper-proof and traceable [23]. The blockchain data structure is shown in Figure 1.

To enhance the efficiency of blockchain traceability queries, Helmer et al. [24] introduced EternityDB, which incorporates database functions into blockchain via smart contracts and transfers databases and data onto the blockchain. However, the large database is replicated across each node in the peer-to-peer network, leading to wasted storage space, increased storage pressure, and higher cost overhead. Muzammal et al. [25] proposed ChainSQL, whose actual data is still stored in the off-chain database. This implies that users must rely on the connected peer nodes and verify the accuracy of the data stored in the database, as query results may not align with the data on the blockchain. Niu et al. [26] developed a new index structure by restructuring the blockchain and integrating prefix tree and ODS technology to enhance the efficiency of transaction searches. This solution enables efficient transaction queries based on transaction hashes and addresses, while also providing privacy protection for resource-constrained clients. He et al. [27] introduced the T-Merkle tree, an innovative blockchain storage structure that enhances the Merkle tree design, boosts query efficiency, and lowers storage costs. Xu et al. [28] introduced the MPT-Chain tree, which merges the benefits of the Patricia tree and the Merkle tree, ensuring distributed index consistency and improving query result accuracy.

### 2.2. Data Traceability Based on Blockchain

Traditional data traceability systems mostly adopt a centralized architecture, but this design is prone to single-point-failure problems, which in turn brings great security risks [29]. In recent years, blockchain technology has provided a highly reliable solution for ensuring data integrity and security with its advantages of decentralization and immutability [30].

Liang et al. proposed ProvChain [31], a data traceability system that leverages blockchain technology. The system maintains data source records through a Merkle tree structure [32]. The data source records can be made public globally and form immutable records after verification. Zhang et al. [33] proposed an efficient and secure data source traceability solution and implemented it in the ESP system. ESP leverages a blockchain-based record chain to offer a secure and efficient platform for data outsourcing, ensuring the accuracy, integrity, and timeliness of data provenance records. The SmartProvenance system, proposed by Ramachandran et al. [34], addresses the shortcomings of ProvChain. It incorporates an automated verification mechanism that effectively prevents potential collusion between external parties and users. The system uses OPM to represent the source record of the data, and records in detail the corresponding user information before and after the data status changes, enhancing the transparency and credibility of data traceability. Griggs et al. [35] created a smart contract system using the Ethereum private blockchain for the analysis and management of medical sensors, enabling secure communication between sensors and smart devices. Porkodi et al. [36] proposed an IoT device management approach based on HABE, enabling the integration of access control policies through the registration and authentication of all users, devices, and sensors within the IoT network. Shahzad et al. [37] proposed a smart grid system design that combines blockchain and the Internet of Things. They used the immutability and transparency of blockchain to improve the security and privacy protection of the system, and used encrypted pseudonyms to replace real identities to prevent malicious entities from obtaining personal privacy information.

### 2.3. Multimedia Security Research in CIoT

With the widespread adoption and application of CIoT devices, security issues, especially in multimedia data transmission and storage, have become increasingly critical. Studies show that CIoT devices often have multiple security vulnerabilities, threatening their security and privacy protection. To address this challenge, many studies have proposed solutions based on advanced technologies. Dimitrakos et al. [38] proposed a zero-trust model combining continuous authorization and trust-aware mechanisms, enhancing data security and privacy protection in CIoT environments. Additionally, Namakshenas et al. [39] explored a quantum computing-based privacy-preserving threat detection model, offering a new approach for secure monitoring of multimedia data. Meanwhile, Williams et al. [40] proposed a scalable approach to identify security vulnerabilities in CIoT devices, providing valuable insights for future multimedia data protection. Recent research by Singh et al. [41] proposed an adaptive and context-aware authentication framework for future vehicular networks that integrates edge AI with blockchain technology, demonstrating the potential of combining decentralized architectures with intelligent authentication mechanisms for multimedia security in distributed environments. These studies show the trend of combining blockchain with other technologies for multimedia security. Our work extends this by optimizing traceability performance for high-volume CIoT multimedia data.

In summary, while existing research has made significant progress in blockchain technology, data traceability, and CIoT multimedia security, there remains a notable gap in addressing the specific requirements of high-performance multimedia data traceability in CIoT environments. Most existing solutions either focus on general data traceability without multimedia-specific optimizations, or address security aspects without providing efficient query mechanisms for large-scale multimedia data. The proposed work aims to bridge this gap by combining the security benefits of blockchain with specialized indexing structures optimized for CIoT multimedia applications.

## 3. Efficient Traceability Mechanism for Multimedia Data Security in CIoT Combined with Blockchain

### 3.1. Blockchain Efficient Traceability Model for Multimedia Data

**Figure 2:**
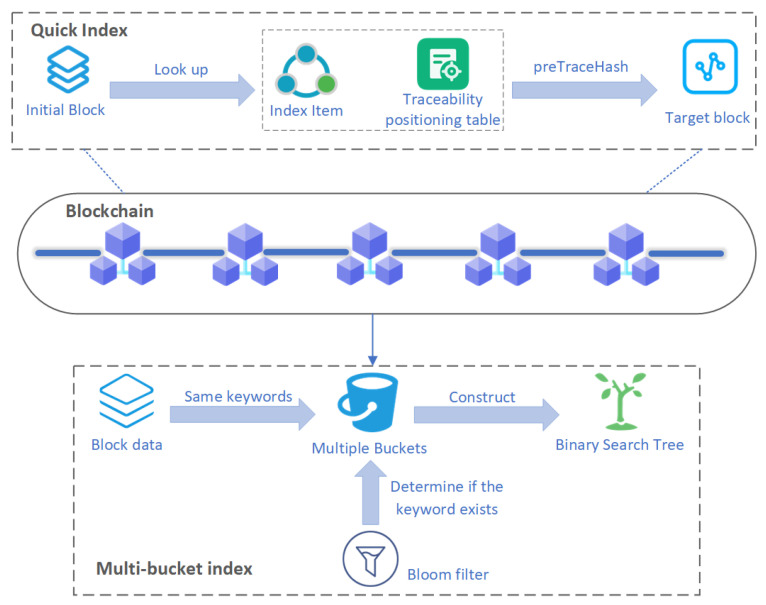
Efficient tracing framework based on joint index.

In traditional blockchain systems, the Merkle tree structure is used to store and verify data. However, due to the decentralized and unordered nature of blockchain data storage, queries often require traversing multiple blocks, resulting in low efficiency. This issue becomes more prominent in scenarios involving large-scale multimedia data and high-frequency queries, where performance bottlenecks hinder effective data access. To address this challenge, this paper proposes an efficient blockchain traceability method based on a joint index structure, targeting multimedia security and big data management in CIoT applications. The proposed method enables fast location of target blocks, avoiding the inefficiency of sequential block traversal and supporting rapid access to transaction data within blocks. As shown in Figure 2, the system model consists of three main components: a blockchain-based PROV model, a fast index structure, and a multi-bucket index structure.

The blockchain-based PROV model provides a standard method for recording data operations, which is employed to record and monitor the entire data operation process, ensuring that the flow of each data object from generation to change can be clearly traced and verified.

The joint index includes fast index and multi-bucket index. The fast index can find the latest information of the target block through the traceability positioning table, and can quickly locate the target block by using the preTraceHash field to obtain complete traceability link information, avoiding the inefficient operation of sequentially traversing blocks.

After the target block is quickly located, the multi-bucket index further optimizes the storage and management of transaction data within the block. The multi-bucket index groups and stores data objects within the block according to keywords, and puts data with the same keyword into the same bucket. This grouping method effectively reduces the amount of data that needs to be scanned during querying, improving query efficiency. The data objects in each bucket are managed through a self-balancing binary search tree, thereby ensuring the efficiency of query operations. When processing large-scale data, the self-balancing tree ensures a query time complexity of O(logn), delivering consistent query performance even with large datasets.

### 3.2. PROV Security Model Based on Blockchain

In traditional blockchain systems, data recording is typically limited to value updates or state changes of transactions, focusing on core details such as inputs, outputs, participants, and timestamps. However, this approach often overlooks the operational context and execution details behind the transactions. In CIoT scenarios, interactions between devices and sensors are highly complex. Data generation, transmission, processing, and storage involve multiple entities, cross-block operations, and lifecycle management. Although transaction data is transparent and tamper-resistant, descriptions of data operations, change processes, and responsible entities are often insufficient. This simplified recording method fails to provide adequate semantic information and traceability in complex CIoT environments, particularly in cases involving multiple parties, cross-block operations, and full data lifecycle tracking. As a result, the potential of blockchain technology in such applications is significantly limited.

The PROV model provides a standard method for recording data operations, and its core components include Entity, Activity and Agent. Among them, each device, sensor or data object can be regarded as an “entity”. Entity not only contains the status data of the device itself, but also includes relationship information with other devices or systems, such as the creator of the device, the source of data collection or previous operation records. Activity in the PROV model is used to describe the specific process related to data operations, including data generation and update operations. Agent is used to identify the participants who perform data operations. Transactions in the blockchain can be jointly participated in by multiple subjects, such as users, smart contracts, systems, etc. By adding subject information to the record of each transaction, the initiator or executor of each data operation can be clearly identified, thereby providing transparency of data operations.

However, the PROV model itself cannot ensure the credibility of data and user identity. To solve this problem, we combine blockchain technology with the PROV model. Specifically, the blockchain distributes data on multiple nodes through a distributed network. Each node stores a copy of the data and maintains its consistency and integrity through a consensus mechanism. Even if some nodes are attacked or fail, other nodes can still maintain the correctness and integrity of the data. In addition, each block in the blockchain is linked to the previous one using a hash algorithm, ensuring data immutability. To address the user identity authentication issue, the blockchain employs the elliptic curve cryptography (ECC) algorithm to generate a pair of public and private keys. The public key uniquely identifies the user, while the private key is used to generate digital signatures, ensuring the immutability and authenticity of transaction or operation records. Whenever a user initiates a transaction or operation, their private key signs the relevant data to form an unforgeable digital signature, which effectively verifies the user’s identity in the data tracing step, ensuring the authenticity and security of the operation.

The proposed mechanism offers comprehensive protection against multimedia content tampering through multiple layers of security. Cryptographic hashing ensures data integrity at the block level, while digital signatures authenticate the source of multimedia operations. The immutable nature of blockchain prevents any modification of historical data, and the PROV model provides detailed audit trails for all multimedia operations. Together, these layers effectively address key security challenges in CIoT multimedia environments, including unauthorized distribution, content tampering, and copyright infringement.

### 3.3. Quick Index

#### 3.3.1. Traceability Positioning Table

In traditional blockchain systems, the Merkle tree structure is used to store and verify data. However, due to the decentralized and unordered nature of blockchain data storage, queries typically require traversing multiple blocks, leading to low query efficiency. This issue becomes particularly pronounced in large-scale energy data and high-frequency query scenarios, where the performance bottleneck of traditional blockchain queries poses a significant challenge. To address this problem, this paper proposes an efficient blockchain traceability method based on a joint index structure, targeting multimedia security and big data management in CIoT applications. This method enables fast location of the target block, avoiding inefficient operations caused by sequential block traversal, thus achieving fast querying of transaction data within the block. The system model of this approach, as shown in Figure 2, consists of three main components: the blockchain-based PROV model, the fast index structure, and the multi-bucket index structure.

In the blockchain system, each object or entity is assigned a unique keyword to distinguish and track the entire life cycle of the transaction. This keyword can be a specific code, hash value, or other form of unique identification. In the index structure proposed in this paper, the keyword “k” is used to identify and associate specific objects. For example, in an asset traceability scenario, related transactions of an asset may share the same keyword, thereby establishing an association between multiple transactions.

The traceability positioning table is the core component of the fast index structure, which is used to efficiently manage and quickly locate the latest block information of the traceability target to ensure its security and consistency. The fast traceability positioning table provides instant access to the target block through the index mechanism, as shown in Equation (1):

(1) $$RTT=\{\left(k_{i},t_{i},h_{i}\right)|i\in \left[1,M\right]\}$$

Here, $k_{i}$ is the keyword of the data object, usually the identifier of the data item, and $t_{i}$ represents the timestamp, which is used to identify the generation time of the data record. $h_{i}$ represents preTraceHash, which is the hash value of the block containing the previous data containing the keyword, and is used to achieve cross-block jumps. *M* is the total number of traceability targets recorded in the traceability positioning table.

**Figure 3:**
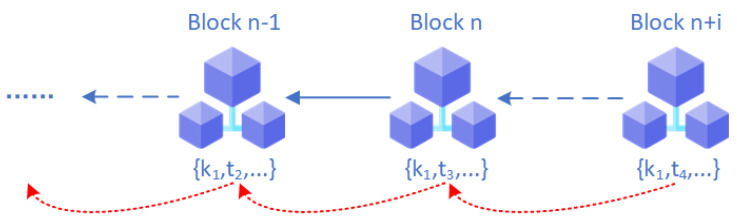
Jumping between blocks.

The structure of the traceability positioning table is shown in Table 1. By giving the keyword, timestamp and preTraceHash field of the traceability target, the location of the target block can be directly located without traversing the entire blockchain, effectively improving the response speed of the blockchain when performing traceability queries. When a new transaction is written to the blockchain, the traceability positioning table will dynamically update the timestamp and preTraceHash of the corresponding traceability target to ensure that these fields point to the latest block information. When a user queries a traceability target, the system first searches in the fast traceability positioning table based on the provided keywords. Once the corresponding record is found, the timestamp and preTraceHash of the target can be obtained to locate the block where the target is located. At this point, the preliminary path of the query has been determined, but to grasp the complete history of the traceability target, it is necessary to recursively jump layer by layer through the preTraceHash field to find the previous related block information until the complete path of the traceability link is obtained. The query path is shown in Equation (2):

(2) $$P_{\mathrm{trace}}\left(h\right)=\left\{\begin{array}{cc} ⌀, & \mathrm{if}\;h=NULL\\ h\cup P_{\mathrm{trace}}\left(p\left(h\right)\right), & \mathrm{if}\;h\neq NULL \end{array}\right.$$

where *h* represents the hash value of the current block, and p(h) represents the preTraceHash in the current block, pointing to the previous related block. In the design of the fast traceability positioning table, the recursive jump of the query path not only speeds up the process of blockchain traceability query, but also ensures the accuracy and timeliness of the query results, as shown in Figure 3. Based on the given keywords, the system can quickly locate the target block, and the preTraceHash field can achieve efficient jumps across blocks, effectively avoiding the inefficient operation of relying on sequential traversal of all blocks in traditional blockchain queries.

To address the issue of delayed updates in the traceability table caused by block reorganization or forks in the blockchain system, this solution designs an automatic compensation mechanism, which automatically triggers the update operation of the traceability table in an event-driven manner when the system detects a change in the blockchain state. Specifically, when the block is rolled back, the system identifies the discarded block (i.e., the “orphan block”) by monitoring the change in the blockchain state. Once a block is detected to be discarded, the traceability table will roll back the impact of the transaction related to the block based on the corresponding transaction record or event log to ensure that the record in the table points to the latest valid block. Additionally, to prevent the loss of key historical data, the system incorporates a rollback log during the update operation of the traceability table. The log records the historical information of all data updates and revocation operations, ensuring that manual or automatic compensation can be performed through the rollback log when anomalies or data inconsistencies occur. The rollback log provides the system with a traceable operation record, so that when an error occurs, it can quickly locate and restore to an accurate state, thereby ensuring that the traceability table can still maintain consistency and integrity in the face of blockchain forks or reorganizations.

The proposed indexing mechanism enhances traceability accuracy by fundamentally reducing two prevalent error sources in traditional blockchain queries: (1) lookup errors due to incomplete or incorrect traversal paths, and (2) stale references caused by delayed updates or chain reorganizations. By enabling direct, hash-based jumps to the latest verified block via the traceability positioning table, the system ensures that queries always retrieve the most current and consensus-validated data version. This eliminates inaccuracies arising from following outdated pointers or missing intermediate state changes, thereby providing a more precise and reliable audit trail for multimedia data provenance.

#### 3.3.2. Comprehensive Path Quantitative Scoring

As the volume of blockchain data grows and query frequency increases, traditional query methods often result in long query paths, negatively impacting query efficiency and response time. This solution optimizes the positioning of the query path through a fast index structure, but during the query process, the quality of the path still directly affects the performance and accuracy of the query. For this reason, a comprehensive scoring mechanism combining path length, block confirmation number, and node reputation is proposed to quantitatively evaluate the blocks in the path and quantify the reliability and security of the path.

Specifically, path length is an important factor affecting query efficiency. The longer the path length, the more complex the operations such as jumps and data verification that need to be performed during the query process, which leads to a decrease in query efficiency. Therefore, the length of the path can be used as a penalty factor to give priority to shorter and more efficient paths to avoid performance bottlenecks caused by lengthy paths. Assuming that the length of the path is *L*, the calculation of the penalty factor $P_{L}$ is shown in Equation (3):

(3) $$P_{L}=\frac{1}{L+\lambda }$$

where λ is a positive adjustment factor (typically set to 1) that prevents division by zero and controls the penalty intensity for longer paths. The number of block confirmations refers to how many times a block is verified by subsequent blocks, which reflects the degree to which the block is verified and accepted in the blockchain network. By accumulating more confirmations, the data of the block is supported by a wider consensus, ensuring that it is not easily attacked or tampered with. Therefore, the number of block confirmations can be used to measure the reliability and security of the path. Assuming that the number of confirmations of the *i*-th block is ${Conf}_{i}$, the confirmation score is shown in Equation (4):

(4) $$Conf_{path}=\sum_{i=1}^{L}Conf_{i}$$

The node reputation score evaluates the credibility of nodes in the blockchain network based on their historical behavior and performance, including their frequency of participating in consensus, success rate of transaction submission, online stability, etc. The reputation score of the path is calculated by weighted average, as shown in Equation (5):

(5) $$R_{path}=\frac{1}{L}\sum_{j=1}^{L}\frac{1}{1+e^{-\alpha_{j}\cdot \left(S_{j}-T\right)}}$$

where *T* represents the reputation score threshold, usually set as the average of historical node behavior; $\alpha_{j}$ is the reputation adjustment coefficient for the *j*-th node, ranging from (0, 1], which controls the sensitivity of the reputation score; and *e* is the base of the natural logarithm. $S_{j}$ represents the reputation base value of the *j*th node, *T* is the threshold of the reputation score, which is used to distinguish nodes with good and poor reputation, *e* is the base of the natural logarithm, and $a_{j}$ is the coefficient for adjusting the node reputation score. In the quantitative scoring of the comprehensive path, $w_{L}$, $w_{C}$, and $w_{R}$ represent the weights of the path length, the number of block confirmations, and the node reputation score, respectively. The weights of each dimension can be dynamically adjusted according to the real-time needs and priority of the query, as shown in Equation (6):

(6) $$S_{path}=w_{L}\cdot P_{L}+w_{C}\cdot Conf_{path}+w_{R}\cdot R_{path}$$

The scoring framework is theoretically grounded in multi-criteria decision theory, with three core components: path length (efficiency), block confirmations (security), and node reputation (reliability). The implementation uses these scores to prioritize query paths and inform caching strategies.

### 3.4. Multi-Bucket Index Structure and Data Query Optimization Within Blocks

#### 3.4.1. Multi-Bucket Index Design

**Figure 4:**
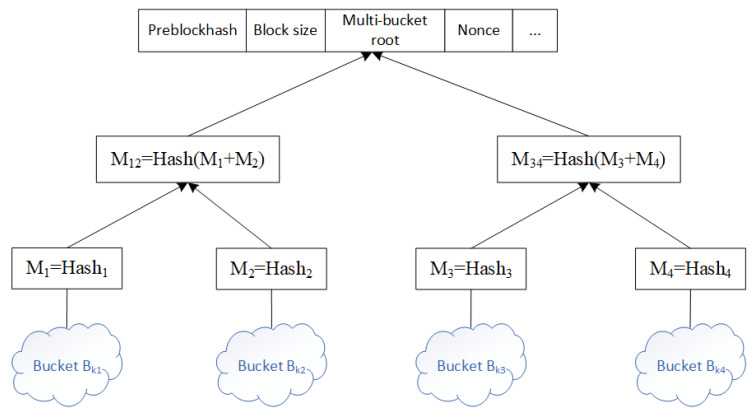
Multi-barrel index.

The multi-bucket index is designed to address the inefficiency of traditional blockchain traceability information storage. In CIoT, data typically comes from multiple devices and sensors and is often structured using Merkle trees. While Merkle trees provide fast verification, queries still require traversing all data nodes in the block, leading to inefficiency. This problem becomes even more pronounced when traceability information is stored across multiple blocks, resulting in higher query costs. Additionally, blockchain storage is usually unordered, further complicating the retrieval of specific keyword data. To address this, this paper designs a multi-bucket index, which stores data in groups within blocks based on keywords and classifies data objects with the same keyword into the same bucket, as shown in Figure 4. The multi-bucket index optimizes query efficiency while maintaining data integrity verification. This design effectively addresses the challenges of large-scale data and high-frequency queries in CIoT, providing strong support for data management and security in blockchain-based CIoT scenarios.

The data in the block is grouped and stored in multiple buckets, each of which corresponds to a specific keyword. A bucket $B_{k_{i}}$ is a collection of data objects under the same keyword $k_{i}$, which is convenient for quick location and organization by keyword, as shown in Equation (7):

(7) $$B_{k_{i}}=\left\{o_{1},o_{2},\ldots ,o_{n}\right\},\mathrm{where}\;o_{i}=\left\{k_{i},v_{i},a_{i},t_{i},h_{i},sigu_{i}\right\}$$

For each data object $o_{i}$, $k_{i}$ is used as a keyword, $v_{1}i$ is the version number, and the historical change information of the data is recorded. $a_{i}$ is the activity related to the data object, $t_{i}$ is the timestamp of the activity, $h_{i}$ represents preTraceHash, and $sigu_{i}$ is the blockchain node signature that executes the activity, which is used to verify the source of the data.

#### 3.4.2. Bloom Filter-Based Screening Mechanism

The data in the block is bucketed according to the keyword, and each bucket contains multiple versions of data items. In order to avoid unnecessary traversal operations, this paper maintains a Bloom filter to record the set of version numbers contained in the bucket. The Bloom filter uses multiple hash functions to ensure that the elements can be evenly distributed in the bit array. The size of the bit array *q* is determined according to the number of data items in the bucket and the expected false positive rate.

When querying, the Bloom filter can quickly determine whether the bucket contains the required version of data, thereby avoiding traversing all data items in the bucket one by one. Assume that there are *n* data items in the bucket, corresponding to *n* version numbers, and the false positive rate *p* is the probability of misjudgment that the system can tolerate, usually a very low value such as 1% or lower. The calculation of the bit array size *q* is shown in Equation (8):

(8) $$q=-\frac{n\cdot \ln p}{{\left(\ln2\right)}^{2}}$$

Bloom filters can quickly determine whether a certain version of a data item exists in the corresponding bucket in the block, thereby avoiding unnecessary traversal operations by users. When the Bloom filter confirms that the data item of the version does not exist during the query, the block can be skipped directly, reducing the query overhead. When a new data object is inserted, the Bloom filter is updated according to the version of the data item, the subscript is obtained through a series of hash functions, and the subscript value is marked from 0 to 1.

Bloom filters may make misjudgments when querying; that is, they may mistakenly return that an element exists, but they will not miss it. Therefore, only when the Bloom filter returns existence, further verification is required to perform precise queries on specific buckets to avoid false positive errors. To reduce false positives, the Bloom filter design must be adjusted to meet the system’s needs. The false positive rate can be effectively minimized by increasing the bit array size or the number of hash functions. Through pre-screening, Bloom filters can effectively reduce the burden on the system and improve query response speed.

#### 3.4.3. Data Management and Self-Balancing Optimization Based on Binary Search Tree

**Figure 5:**
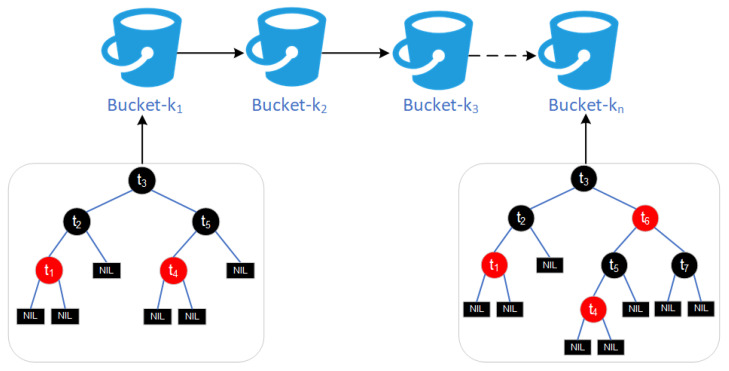
Self-balancing binary search tree.

For scenarios with large amounts of data in a block, when transaction data is bucketed according to keywords, if it is managed in the form of an ordered table, it will face the problem of low search efficiency. To this end, for the data object set under the same keyword *k*, this paper uses a self-balancing binary search tree to manage it, as shown in Figure 5. Each node of the binary search tree represents a specific data object, and its structure contains multiple fields to support ordered storage and fast access, as shown in Equation (9):

(9) node={sort,ptr,color,left,right,parent}

Among them, the sort key sort is used to maintain the orderliness of the binary search tree. In this design, the timestamp $t_{i}$ of the data object is selected as the sort key to ensure the orderly arrangement of the data object, which is convenient for query and ordered traversal based on the time range. To avoid redundant storage, ptr is a pointer to the transaction data object. The color attribute of the node can only be marked as red or black. The color attribute ensures that the tree remains balanced after inserting and deleting nodes through a series of rules and rotation operations. The left and right pointers point to their left and right child nodes respectively, while parent points to the parent node of the current node, which is used to quickly backtrack and locate nodes when adjusting the tree structure (such as rotation operations).

In order to ensure the self-balancing property of the binary search tree, the following basic properties are stipulated; that is, each node of the tree can only be red and black, and the root node and all leaf nodes (i.e., NIL nodes) are black. In addition, the path from any node to all its leaf nodes must contain the same number of black nodes. The child nodes and parent nodes of red nodes are all black, thus preventing the height of the tree from being too high.

When each bucket $B_{k}$ is initialized, an empty tree structure is created. The construction of the tree starts from the root node, and its structure is gradually expanded as data objects are continuously inserted. Initially, the root node pointer of the tree points to a black NIL node, indicating the terminal of the tree. The root node itself does not store any actual data objects. The root node will not be assigned a value until the first data object is inserted, and its color is marked as black. As data objects are inserted, the binary search tree maintains its balance and order by adjusting the node color and performing necessary rotation operations.

Specifically, when subsequent transaction data is continuously inserted, the system continues to determine the bucket to which it belongs based on the keyword of the transaction data, and finds a suitable position in the corresponding binary search tree for insertion. Although the data is first assigned to different buckets based on the keyword, within each bucket, the binary search tree is sorted according to the timestamp of the data object. When new transaction data is inserted as a new node, its appropriate position in the tree is determined based on the timestamp ti of the data object, and ti is compared with the key value of the current node layer by layer to decide whether to move to the left subtree or the right subtree until an empty NIL node is found as the insertion point. Once the insertion position is determined, the new node is created and assigned a red color. If the insertion causes the properties of the tree to be destroyed, such as the appearance of consecutive red nodes, the system adjusts it through color flipping and rotation operations to restore the balance and red-black properties of the tree. The specific adjustment steps include: if the parent node is black, no adjustment is required; if the parent node is red and the uncle node is also red, color flipping is performed; if the parent node is red but the uncle node is black or does not exist, the structure of the tree is adjusted and recolored through left or right rotation operations. Through the above color adjustment and rotation operations, the binary search tree can be rebalanced after inserting a new node, ensuring that its basic properties are not destroyed, thereby maintaining the tree’s high balance and operational efficiency.

When the parent node and uncle node of the newly inserted transaction data are both red, in order to maintain the balance of the tree, it is usually necessary to change the parent node and uncle node to black, and change the grandparent node to red. However, the color change of the grandparent node may trigger conflicts at a higher level, resulting in recursive adjustments to the root node of the tree, increasing the time complexity and space complexity of the insertion operation. To this end, this paper designs an instant adjustment algorithm, as shown in Algorithm 1. Here, node is the grandparent node of the newly inserted transaction data. When it is detected that the parent node and uncle node of the newly inserted data are both red, the algorithm will immediately make adjustments at the current level, instead of recursively moving the problem to a higher level and waiting until the node insertion is completed before making recursive corrections. The instant adjustment algorithm can ensure that each adjustment operation is limited to the current node and its directly associated parent node and uncle node, avoiding deep recursion in the worst case, and limiting the scope of operations to constant time.
**Algorithm 1** Timely Adjustment1:**Input:** *tree*, *node*2:**Output:** local adjustments to the tree structure3:**function** TimelyAdjustment(*tree*, *node*)4:      **if** *node.left* ≠ null **and** *node.right*≠ null **then**5:            **if** *node.left.color* = red **and** *node.right.color* = red **then**6:                 *node.left.color* ← black7:                 *node.right.color* ← black8:                 *node.color* ← red9:                 **if** *node.parent* ≠ null **and** *node.parent.color* = red **then**10:                       run *fixup(tree, node)*11:                **end if**12:           **end if**13:     **end if**14:**end function**

In the process of querying data objects, precise query starts from the root node of the binary search tree, compares the target timestamp $t_{i}$ with the key value of the current node layer by layer, and decides to move to the left subtree or the right subtree until a matching node is located or the target data is confirmed to be non-existent. This process uses the self-balancing characteristics of the binary search tree to ensure that the time complexity of the query operation is maintained at O(logn), where *n* is the number of nodes in the tree, ensuring efficient single data object retrieval. In addition, its unique balancing mechanism avoids excessive growth of the tree height, thereby effectively ensuring the efficiency of the query operation. Compared with the traditional linear linked list structure or non-self-balancing binary search tree, this balancing mechanism ensures that the query path always remains within the optimal range, thereby reducing unnecessary comparison operations and further improving the search speed. Especially in the scenario of dynamic data update, the structure of the tree can be adjusted adaptively, ensuring that the time complexity of the balance adjustment operation when inserting and deleting nodes is also maintained at O(logn), so that the query efficiency remains stable.

### 3.5. Security Analysis and Risk Mitigation

To provide a systematic and structured security analysis, we categorize and address the key security risks identified in our proposed system:

- Keyword Pattern Exposure Protection: Keywords are generated using cryptographic hashing of content identifiers combined with device-specific metadata, ensuring that patterns cannot be reconstructed from observable data. The hash-based approach prevents correlation attacks and maintains data privacy while enabling efficient indexing.
- Secure Key Management for CIoT Devices: We employ elliptic curve cryptography (ECC) for digital signatures, which provides strong security with relatively small key sizes suitable for resource-constrained CIoT devices. The system implements a lightweight key management protocol that includes periodic key rotation and secure key storage mechanisms, balancing security requirements with device limitations.
- Resilience to Prolonged Forks: Our traceability positioning table incorporates an automatic compensation mechanism with rollback logs that ensure consistency during blockchain reorganizations. This design maintains data integrity and query accuracy even during extended fork scenarios, preventing stale references and ensuring reliable traceability.
- Index Security and Tamper Resistance: All index structures are protected through the same cryptographic mechanisms as the blockchain data itself. Any attempt to modify index entries would require breaking the blockchain’s consensus, ensuring that indexes maintain the same immutability properties as the underlying data.
- Sybil Attack Resistance: The node reputation scoring system integrated into our path evaluation mechanism helps mitigate Sybil attacks by prioritizing queries through nodes with established trust histories, making it economically and computationally impractical for attackers to manipulate the traceability system.

These security considerations are integral to our design and have been implemented throughout the proposed mechanism. The combination of cryptographic protections, structural resilience, and trust management provides comprehensive security coverage for CIoT multimedia traceability applications.

### 3.6. Summary of Model Robustness and Security

The proposed mechanism is designed with CIoT constraints in mind. The keyword management (Section 3.3) uses cryptographic hashing for uniqueness and integrity. The PROV model integration (Section 3.2) is extended with event aggregation for handling high-frequency CIoT data streams. The self-balancing tree and fork compensation algorithms ensure data structure stability under concurrent updates. A comprehensive security analysis addressing index protection, pattern obfuscation, and Sybil attack resilience is inherent to the blockchain-PROV foundation and the reputation-based scoring system for reproducibility.

## 4. Simulation Experiment and Analysis

To evaluate the feasibility and performance of the efficient blockchain tracing method based on the joint index structure proposed in this paper, this section designs relevant experiments to thoroughly assess the scheme’s performance during block creation and traceability queries. The experimental environment is as follows: CPU: Intel(R) Core(TM) i7-12700H CPU @ 3.50 GHz, memory: 16 GB, hard disk: 1 TB. In addition, this experiment uses the BlockChainDemo blockchain system to simulate 1–10 source information for each original data to simulate the relevant operations in the blockchain network.

Given the increasing importance of CIoT applications, which involve large-scale data and real-time multimedia interactions between devices, the primary focus of this experiment is to evaluate query performance in high-frequency, high-data-volume environments. This is closely related to the challenges faced in CIoT applications, such as multimedia security and big data management, where query efficiency becomes a critical issue.

The core goal of this experiment is to deeply explore the significant improvement in query efficiency brought by the joint index structure when processing large-scale data sets through comparative analysis. To comprehensively assess the performance benefits of the joint index structure, the experimental design covers four main evaluation indicators: block construction time, the impact of block size and different data scales on query time, and the number of records traversed when querying specific transaction data.

We acknowledge that the current experiments, conducted on a local simulator, primarily validate the algorithmic efficiency of our indexing structures. The significant performance gains demonstrated (e.g., 60–75% reduction in query time) establish a strong theoretical foundation. Future work will focus on deploying the system in a distributed CIoT testbed to evaluate performance under real-world network conditions, resource constraints, and device heterogeneity, as outlined in Section 5.

### 4.1. Build Time Overhead

**Figure 6:**
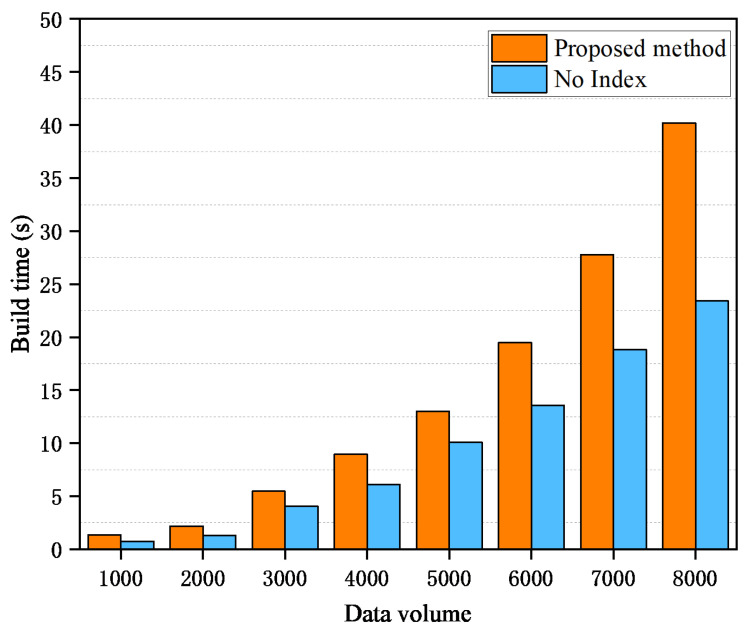
Block construction time overhead with different index structures.

The block construction overhead is mainly used to measure the time required to generate a block under different index structures, to reflect the construction efficiency of the blockchain system. The experimental results for block construction time are presented in Figure 6. As the data scale increases, the block construction time using the joint index structure shows a corresponding time overhead compared with the traditional method. This can be attributed to the fact that the joint index design requires additional maintenance of metadata for two index structures: fast index and multi-bucket index. Specifically, the fast index avoids unnecessary traversal operations by maintaining the traceability positioning table and storing cross-block jump information (such as the preTraceHash field), thereby improving the query efficiency. However, this process requires frequent updates to the traceability table, which increases the computational complexity of the block construction process. In addition, the multi-bucket index groups transaction data within the block and uses a self-balancing binary search tree to efficiently manage the data in each bucket. Although the introduction of this structure improves query efficiency, it also brings additional time overhead to block construction, especially when the data volume is large, and the maintenance cost of the self-balancing tree in the bucket cannot be ignored. Although the joint index structure introduces additional overhead during block construction, it is essential to evaluate this cost against the substantial query-time savings it enables. The maintenance cost primarily involves updating the traceability positioning table and rebalancing the binary search trees within buckets upon new data insertion. However, as demonstrated in Section 4.2, the query efficiency gains-evident in the significantly lower growth rate of query time and reduced number of traversed records-far outweigh these incremental update costs, especially in CIoT environments characterized by high-frequency read operations. The synchronization of index metadata across blockchain nodes is efficiently managed through the underlying consensus protocol, ensuring consistency without compromising the system’s decentralized security guarantees.

This additional construction overhead represents the quantifiable cost of enabling fast queries. Crucially, as shown in Section 4.2, this cost is offset by an order-of-magnitude improvement in query efficiency and a dramatic reduction (60–75%) in the number of records traversed. The index synchronization overhead is managed by piggybacking on the blockchain’s native consensus protocol, ensuring consistency without separate complex mechanisms.

### 4.2. Query Performance Test

In this section, we begin by conducting a query time test on the proposed joint indexing scheme to examine how block size affects query efficiency. Then, we compare the proposed scheme with traditional methods and the SEBDB [16] method. We evaluate the performance of querying specific transaction data and the number of records that need to be traversed during the query process under different data scales through simulation experiments.

The joint index structure in this scheme avoids the inefficient operation of sequential traversal of blocks by combining fast indexing and multi-bucket indexing. Among them, fast indexing realizes direct jumps across blocks through the preTraceHash field, which greatly reduces the overhead of block traversal during the query process. Multi-bucket indexing further optimizes the query efficiency within a single block by grouping data within the block and managing the data in the bucket using a binary search tree structure. In order to evaluate the performance overhead of the proposed scheme in traceability queries, this section designs experiments under different block sizes and data scales, and compares and analyzes the performance of the proposed scheme under different conditions.

**Figure 7:**
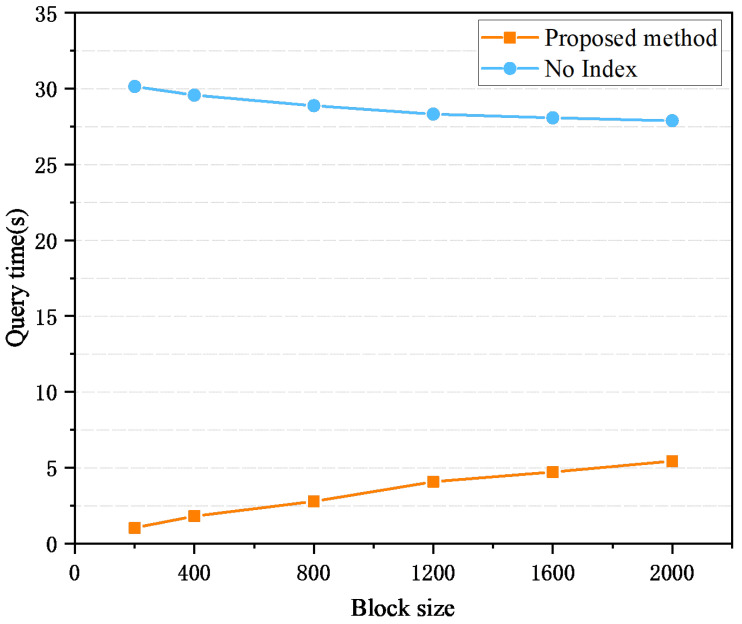
Query time efficiency under different block sizes.

First, this section examines the effect of block size on query efficiency. The experimental results are displayed in Figure 7. The figure shows that when the block size is the same, the query time of the joint index is significantly shorter than that of the traditional blockchain, highlighting the efficiency of the solution in this article. However, as the block size grows, the query efficiency of the traditional blockchain is improved, while the time overhead of the joint index query gradually increases. This is because the traceability query of the traditional blockchain relies on linear traversal of each block. When the amount of transaction data remains constant and the block size increases, the total number of blocks decreases, which in turn reduces the number of blocks that need to be traversed during the query process. The query efficiency of the traditional blockchain is improved under the condition of larger blocks. In contrast, as the block size increases and the total number of blocks decreases, the need for block jumps also gradually decreases, diminishing the advantage of the joint index in jump optimization.

**Figure 8:**
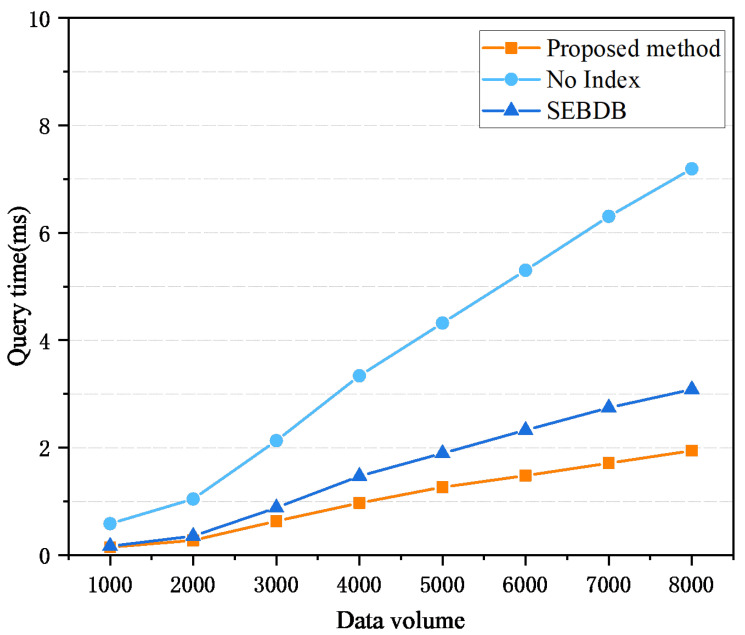
Query time under different data volumes.

Then, this section studies the traceability query time under different data scales, and the experimental results are shown in Figure 8. It is evident from the figure that as the data scale continues to increase, the query time of the traditional index structure shows a rapid growth trend. The query time of SEBDB increases with the data scale, but the rate of increase is significantly smaller compared to the traditional method. The joint index structure shows a relatively stable growth rate, which is better than the above two methods and has the best performance. The experimental results reflect the significant advantages of the joint index structure in optimizing query efficiency, especially in dealing with large-scale data scenarios.

The traditional blockchain query method requires traversing the data block by block and searching for the target transaction through linear scanning. This method has no obvious impact in a small-scale data environment, but as the data scale increases, its query time increases exponentially, resulting in performance bottlenecks. The main optimization of SEBDB is the combination of database and blockchain data. Although the query logic is simplified through the SQL-like interface, its query time will gradually increase when dealing with large data volumes and high-frequency queries. In contrast, the advantages of the joint index gradually emerge as the data scale continues to expand. Its query time overhead is much smaller than that of the traditional method and has good scalability.

**Figure 9:**
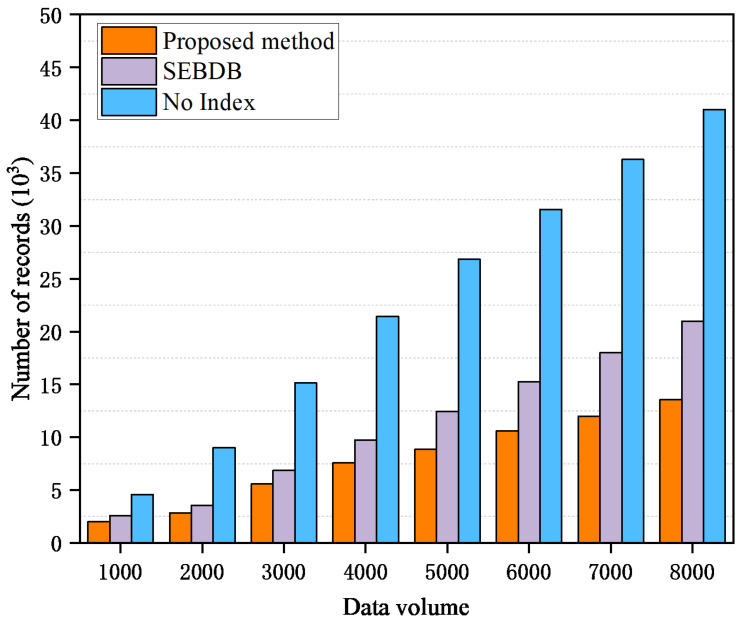
Number of records traversed in queries with different data volumes.

In order to test the number of records that need to be traversed when performing traceability queries for joint indexes, traditional methods, and SEBDB methods under different data scales. The number of records traversed during the query process was tested through simulation experiments to reflect the efficiency of the query operation. If more records need to be traversed, the time spent on the query operation will also increase accordingly. Therefore, reducing the number of records traversed is the key to improving query efficiency. The results of the experiment are presented in Figure 9. It is clear that the traditional blockchain can maintain a certain query efficiency when the data scale is small, but with the increase of data scale, especially from 2000, the number of records that need to be traversed increases significantly. Due to the lack of clear index support, querying a specific transaction requires traversing many irrelevant records, which brings significant time overhead. SEBDB has better performance than traditional methods, but its query performance optimization mainly depends on its hierarchical index, and there is no special optimization for cross-block data queries. It still needs to scan multiple data tables or blocks to a certain extent, so it will expose the problem of traversing a large number of records when querying large-scale data. The performance of the joint index structure in this indicator is relatively stable, and the number of traversed records increases slightly. Through the combination of fast index and multi-bucket index, the traversal of invalid records is greatly reduced, thereby significantly improving the query efficiency. Therefore, although the joint index structure has a relatively high time overhead in the block creation process, it greatly enhances the efficiency of traceability queries. The benefits it brings in performance optimization are huge, showing its superiority.

The superior performance of the proposed joint index structure is driven by several key optimizations. The fast index eliminates the need for sequential block traversal by enabling cross-block jumps, significantly reducing the time required to locate the latest traceability block. Additionally, the multi-bucket index organizes data efficiently, minimizing the search space and enhancing query speed. The use of self-balancing binary trees ensures that query complexity remains optimal even with dynamic data updates. These optimizations are particularly advantageous for CIoT multimedia applications, where real-time responses and high query frequencies are essential.

## 5. Conclusions

This paper proposes an efficient blockchain traceability method using a joint index structure, aiming to address the performance bottlenecks faced by traditional blockchains in large-scale data and high-frequency query scenarios. In CIoT applications, numerous devices, sensors, and real-time data streams are typically involved. Although traditional blockchain data structures ensure data storage and verification, the unordered and decentralized nature of data storage requires traversing multiple blocks during queries, leading to low query efficiency. To address this issue, this paper introduces a joint index structure, consisting of a fast index and a multi-bucket index. The fast index, through the maintained traceability positioning table, quickly locates the target block, enabling cross-block jumps and significantly improving query efficiency. Additionally, the multi-bucket index groups data objects by keyword within the block and uses a self-balancing binary search tree for efficient management. This ensures both high query efficiency and system stability. In conclusion, this approach not only effectively improves blockchain query efficiency but also enhances data traceability. Future research could further explore smart contracts to address the growing query demands in CIoT, extending the application of blockchain technology in CIoT, particularly in multimedia security and big data management.

## Figures and Tables

**Table 1:** Traceability positioning table.

Keywords	Timestamp	preTraceHash
$k_{1}$	$t_{1}$	0x124ghb246…
$k_{1}$	$t_{2}$	0x634brx537…
$k_{2}$	$t_{3}$	0x483dgd709…
$k_{3}$	$t_{5}$	0x638agh971…
…	…	…

## Data Availability

The original contributions presented in this study are included in the article. Further inquiries can be directed to the corresponding author.
